# Deciphering the hematopoietic cell ecosystem

**DOI:** 10.1097/BS9.0000000000000211

**Published:** 2024-11-26

**Authors:** Hui Cheng

**Affiliations:** a State Key Laboratory of Experimental Hematology, National Clinical Research Center for Blood Diseases, Haihe Laboratory of Cell Ecosystem, Institute of Hematology & Blood Diseases Hospital, Chinese Academy of Medical Sciences & Peking Union Medical College, Tianjin 300020, China; b Tianjin Institutes of Health Science, Tianjin 301600, China

The circulatory system is a complex network that serves as a lifeline for nutrient distribution, waste removal, and immune defense. At its core, th e blood ecosystem is a dynamic and finely tuned environment where diverse cellular components coexist and interact to maintain homeostasis.

The blood ecosystem is regulated by a delicate balance between biochemical signals and cellular interactions. Hematopoietic stem cells (HSCs) reside at the apex of this system and give rise to all blood cell lineages. The transition from HSCs to mature blood cells is a tightly orchestrated process involving numerous growth factors, cytokines, and transcription factors that dictate cell fate and differentiation. Moreover, the blood ecosystem extends beyond its cellular components, encompassing the vascular niches that provide a supportive architecture for blood cell development. Bone marrow serves as the main site for hematopoiesis, providing a nurturing microenvironment where HSCs find their niche and balance self-renewal and differentiation. Integrity of the blood ecosystem is crucial for human health. Disruptions can lead to various blood disorders including anemia, leukemia, and coagulation disorders. Understanding the complex interplay within the blood ecosystem is essential for developing treatments and therapies to restore balance and function in cases of dysregulation.

To address this issue, 4 experts were invited to provide insights on the regulation of blood ecosystems: Prof. Haojian Zhang, Prof. Pengxu Qian, Prof. Meng Zhao, and Prof. Yu Lan. All researchers have a strong research background in this field, with specific training and expertise in epigenetics, gut microbiota, megakaryocyte (MK) regulation, hemogenic endothelial cells (HECs), and artificial intelligence. They share unique perspectives on blood ecosystem regulation in hematopoietic development and blood diseases. A brief introduction to their research is as follows:

Prof. Haojian Zhang from Wuhan University focuses on epigenetic and transcriptional regulation in hematopoietic development and blood diseases. He presents a review article in this issue titled “Epigenetic Modifications in the Hematopoietic Ecosystem: A Key Tuner from Homeostasis to Acute Myeloid Leukemia.” This review provides an in-depth analysis of how epigenetic changes, particularly DNA methylation and RNA m6A modifications, serve as pivotal regulators of the transformation of HSCs into leukemia stem cells, leading to acute myeloid leukemia (AML). It elucidates the intricate balance of DNA methylation required for maintaining HSC function and how its dysregulation contributes to the initiation and progression of AML. Additionally, this review also highlights the role of RNA m6A modifications in the self-renewal and differentiation of HSCs and their reprogramming during AML development. This review makes a significant contribution to the field by offering insights into the complex interplay of epigenetics in AML and paving the way for potential therapeutic strategies targeting epigenetic modifications.

Prof. Pengxu Qian from Zhejiang University focuses on the regulation of transcription factors and gut microbiota in the self-renewal and differentiation of HSCs. He has published a review article titled “Gut microbiota plays pivotal roles in benign and malignant hematopoiesis,” which provides up-to-date information on the regulation of HSC homeostasis by the gut microbiota. This review highlights the significant influence of the gut bacteria on blood cell formation and blood-related diseases. It discusses how alterations in the gut microbiota can affect the hematopoietic system, potentially leading to both benign and malignant hematological conditions. Microbiota imbalances have been linked to leukemia, multiple myeloma, and other blood cancers, thereby influencing immune responses and disease progression. This review emphasizes the role of gut microbiota in regulating HSCs and suggests that restoring the microbial balance could improve treatment outcomes for these conditions. It also highlights the therapeutic potential of targeting gut microbes to prevent disease onset and enhance cancer treatment efficacy, signaling an emerging frontier in hematology and oncology.

Prof. Meng Zhao from Sun Yat-sen University focuses on basic and translational research on stem cells and blood disease treatment. Prof. Zhao presents a review article titled “The versatility of megakaryocytes in homeostasis and disease,” which offers a comprehensive summary of the multifaceted roles of MKs in both physiological and pathological conditions. This review shows the intricate processes of megakaryocytopoiesis and thrombopoiesis and highlights the pivotal role of MKs in platelet production for hemostasis and wound healing. It underscores the emerging understanding of heterogeneity of MKs and its dynamic involvement in immune responses and HSC regulation, as well as its implications in diseases such as myelofibrosis and myeloproliferative neoplasms. It also addresses the impact of aging on MK function, suggesting that age-related changes in MKs may contribute to increased thrombosis. This review is a significant contribution to the field, providing a thorough synthesis of current knowledge and potential directions for future research on MKs.

Prof. Yu Lan from Jinan University focuses on lineage patterns and regulatory mechanisms in the development of vascular and hematopoietic systems. She presents a review entitled “New insights into the endothelial origin of the hematopoietic system inspired by ‘TIF’ approaches.” This review explores groundbreaking insights into the endothelial origins of the hematopoietic system, using advanced “TIF” (transcriptome, immunophenotype, function/fate) approaches. Hematopoietic stem and progenitor cells (HSPCs) are derived from HECs through the endothelial-to-hematopoietic transition during embryogenesis. Single-cell technologies and lineage-tracing techniques identify crucial markers and developmental pathways, highlighting the transition stages of HECs and their progenies, including pre-HSCs (precursors of HSCs). These techniques provide detailed molecular and functional insights and identify the key cell populations responsible for hematopoiesis in both mice and humans. Moreover, it elucidates the controversial onset of Kit expression at the HEC stage, enhancing our understanding of this transition. This review also emphasizes the role of specific embryonic regions, such as the aorta-gonad-mesonephros, in the emergence of HSPCs, with applications for improving stem cell therapies. This work represents a significant leap in developmental biology and offers a precise understanding of how blood cells originate from endothelial structures during early development.

